# Spectroscopic and Thermal Characterisation of Interpenetrating Hydrogel Networks (IHNs) Based on Polymethacrylates and Pluronics, and Their Physicochemical Stability under Aqueous Conditions

**DOI:** 10.3390/polym16192796

**Published:** 2024-10-01

**Authors:** David S. Jones, Marion Westwood, Shu Li, Gavin P. Andrews

**Affiliations:** School of Pharmacy, Queen’s University of Belfast, 97, Lisburn Road, Belfast BT9 7BL, UKs.li@qub.ac.uk (S.L.); g.andrews@qub.ac.uk (G.P.A.)

**Keywords:** interpenetrating hydrogel networks (IHNs), biomaterials, spectroscopy, pluronic block copolymers, glass transition, micellization, retention

## Abstract

This study describes the physicochemical characterisation of interpenetrating hydrogel networks (IHNs) composed of either poly(hydroxyethylmethacrylate, p(HEMA)) or poly(methacrylic acid, p(MAA)), and Pluronic block copolymers (grades F127, P123 and L121). IHNs were prepared by mixing the acrylate monomer with Pluronic block copolymers followed by free radical polymerisation. p(HEMA)–Pluronic blends were immiscible, evident from a lack of interaction between the two components (Raman spectroscopy) and the presence of the glass transitions (differential scanning calorimetry, DSC) of the two components. Conversely, IHNs of p(MAA) and each Pluronic were miscible, displaying a single glass transition and secondary bonding between the carbonyl group of p(MAA) and the ether groups in the Pluronic block copolymers (Raman and ATR-FTIR spectroscopy). The effect of storage of the IHNs in Tris buffer on the physical state of each Pluronic and on the loss of Pluronic from the IHNs were studied using DSC and gravimetric analysis, respectively. Pluronic loss from the IHNs was dependent on the grade of Pluronic, time of immersion in Tris buffer, and the nature of the IHN (p(HEMA) or p(MAA)). At equilibrium, the loss was greater from p(HEMA) than from p(MAA) IHNs, whereas increasing ratio of poly(propylene oxide) to poly(ethylene oxide) decreased Pluronic loss. The retention of each Pluronic grade was shown to be primarily due to its micellization; however, hydrogen bonding between Pluronic and p(MAA) (but not p(HEMA)) IHNs contributed to their retention.

## 1. Introduction

Hydrogels are three-dimensional crosslinked hydrophilic polymers that can absorb considerable masses of aqueous fluids through polymer chain expansion and yet maintain their mechanical properties [1]. They can be homopolymers or copolymer networks and are frequently classed according to the nature of their side groups, as either neutral or ionic [2]. The selective choice of acrylate monomers and polymerisation by free radical polymerisation enables the production of polymers that can demonstrate a wide range of physicochemical properties, notably mechanical properties [3], lubricity [4], swelling [5], drug release and degradation [2,6].

Medical-device-associated infections are, unfortunately, the most common nosocomial infection within the primary care setting [7,8], with the incidence of infection being dependent on both the site of implantation and whether the device is external to the body. For example, it has been reported that catheter-associated urinary tract infections account for 9% of all hospital-acquired infections and that approximately two-thirds of these could have been prevented [9]. The annual costs of healthcare-associated infections have been reported to be circa USD 33 billion in the U.S. [10]. Due to their versatility, hydrogels have found application as matrices/coatings for medical devices and as drug delivery systems. Hydrogels are the mainstay of lubricious coatings of urinary catheters, e.g., Lofric and Speedicath [11]. Furthermore, several hydrogel-based approaches have shown promise for the prevention/treatment of medical-device-related infections. These include silver-containing hydrogels [12,13,14], antimicrobial (antibiotics, antimicrobial polymers/peptides) hydrogels [15,16], photosensitisers [17], polyelectrolyte and polyzwitterionic hydrogels [18], and double network chitosan hydrogels [19].

Despite their widespread usage in urology, several limitations are associated with hydrogels as medical device materials. Firstly, their mechanical properties are often inappropriate for fabricating devices solely using these materials, and whilst this may be addressed by, e.g., changing the monomer composition or the degree of crosslinking, these interventions may compromise other properties, e.g., release kinetics of the bioactive component [20], contact angle [20], and catheter lubricity [21]. An approach that may be used to modify the properties of mono-polymeric systems, including hydrogels, is interpenetrating polymer networks, which contain two or more interlaced polymers and may be designed to retain the key functionalities of each component [22]. In this study, we sought to design biomaterial platforms that may be used as medical device biomaterials and/or drug delivery platforms that provide greater functionality. Specifically, we describe interpenetrating hydrogel networks (IHNs) composed of either poly(hydroxyethylmethacrylate, p(HEMA)) or poly(methacrylic acid, p(MAA)) and block copolymers of poly(ethylene oxide) (PEO) and poly(propylene oxide) (PPO) (Pluronics), arranged in a PEO-PPO-PEO triblock structure. The choice of Pluronic block copolymers as the second network component was informed by the reported use of Pluronic F127 as a brush coating for medical devices to reduce subsequent microbial colonisation [23,24] and the adhesion of blood components [25]. Normally, chemical grafting techniques are required to attach the Pluronic block copolymers to the polymeric device; however, the network structures described in this study may be of potential value in this respect, thus avoiding the need for polymer grafting. Furthermore, the networks described herein may offer advantages for improved delivery of bioactive agents, again designed to control medical-device-related infection.

In this study, we describe the physicochemical (spectroscopic and thermal) properties of IHNs to provide a greater understanding of the physical state of and interactions between the two polymer components and how these interactions affect retention within the network when immersed within an aqueous fluid. This provides an invaluable key step in understanding the practical uses of this IHN as medical device biomaterials/coatings.

## 2. Materials and Methods

### 2.1. Chemicals

The materials used were of analytical grade or higher and were used as supplied. 2-Hydroxyethylmethacrlyate 97% (HEMA), methacrylic acid 99% (MAA), benzoyl peroxide, ethylene glycol dimethacrylate 98% (EGDMA), and Pluronic F127 (F127) were purchased from Aldrich, Poole, Dorset, UK. Pluronic P123 (P123) and Pluronic L121 (L121) were supplied by BTC UK, Cheshire, UK. To facilitate sample preparation, medical-grade silicone tubing was purchased from S.F. Medical, Hudson, MA, USA, Poly(ester) film, one-sided siliconised release liner (FL2000TM PET 75 μ 1S) was purchased from Rexam Release B.V., Apeldoorn, The Netherlands, and glass plates (100 mm × 150 mm × 3 mm) were purchased from J.E. Harrison Ltd. Glaziers, Belfast, Northern Ireland.

### 2.2. Synthesis of Network Homopolymers and IHNs

p(HEMA) homopolymer and p(HEMA)/Pluronic interpenetrating hydrogel networks (IHNs) were prepared by initially dissolving the crosslinking agent EGDMA (2% *w*/*w*) and the free radical initiator benzoyl peroxide (1% *w*/*w*) in the required amount of HEMA in a flat-bottomed beaker. The mixture was stirred mechanically until benzoyl peroxide had fully dissolved. The resultant solution was then injected into a mould comprising two vertical glass plates lined with a siliconised release liner, separated by medical-grade tubing and clamped together by bulldog clips. The mould was placed in a fan-assisted oven at 90 °C for two hours to produce the p(HEMA) control. To produce the IHNs, Pluronic block copolymers (L121, P123 or F127) were dissolved in this monomer mixture at defined concentrations (10%, 20%, 30%, 40% *w*/*w*). The maximum concentration of Pluronic block copolymers, which could be dissolved in HEMA, was 40% *w*/*w*. The resultant solution was then polymerised under the same conditions as p(HEMA).

p(MAA)/Pluronic IHNs were prepared similarly; however, a further concentration of Pluronic (50% *w*/*w*) was added to the monomer solution.

### 2.3. Modulated Temperature Differential Scanning Calorimetry (MTDSC) of IHNs

MTDSC experiments were performed using a T.A. Instruments Q100 Modulated Differential Scanning Calorimeter (T.A. Instruments, West Sussex, UK) with a refrigerated cooling system. Small sections (7–10 mg) of each material were cut using a scalpel and placed in an oven at 60 °C for 72 h to remove any unreacted monomer. Heat flow was calibrated using an indium standard, and heat capacity with a sapphire standard. Nitrogen was used as the purge gas, with a flow rate of 50 mLmin^−1^ through the DSC cell. Standard aluminium pans and lids from T.A. Instruments were used throughout the experiments. The pans were sealed using a T.A. Instrument press.

Samples were exposed to an initial 5 min isothermal period at −100 °C followed by a modulation amplitude of ±0.7 °C every 50 s with a 2 °C min^−1^ underlying heating rate to 160 °C for p(HEMA) IHNs and to 250 °C for p(MAA) IHNs. Total heat flow, reversing heat flow and non-reversing heat flow were calculated automatically by the Universal Analysis 2000 software as a function of temperature. Experiments were performed at least in quadruplicate for each candidate material.

The glass transition (T_g_) was taken as the midpoint of the slope change of the heat capacity. The melting temperature (T_m_) was accepted as the minimum of the endothermic peak, whereas the crystallisation temperature (T_c_) was determined as the maximum of the exothermic peak. The heat of melting (ΔH_m_) and the heat of crystallisation (ΔH_c_) were evaluated from the areas of the melting and crystallisation peaks, respectively. The percentage crystallinity, χ_c_, was calculated from Equation (1) [26,27]:

(1) $$\chi_{c\left(PEO\right)=\left(\frac{{\Delta H}_{mPEO}}{w_{cryst}{\Delta H}_{mPEO}^{o}}\right)\times 100}$$

where ΔH_m,PEO_ is the apparent melting enthalpies of PEO present in the network, w_cryst_ is the weight fraction of the crystalline component in the blend and ΔH_mPEO_^0^ is the heat of melting per gram of 100% crystalline PEO. It has been reported to be 188 kJ Kg^−1^ [28].

### 2.4. Dynamic Mechanical Analysis (DMA) of IHNs

DMA was performed using a Dynamic Mechanical Analyser model 2980, (T.A. Instruments). The machine was calibrated for position, force, and electronics every month. Samples of approximate dimensions 30 mm × 10 mm were cut from the films using a scalpel. Using single-cantilever clamps, the sample was subjected to a sinusoidal oscillation at a frequency of 1 Hz while the sample was heated at a rate of 2 °C min^−1^ from 30 °C to 200 °C. The storage modulus and loss tangent (tan δ) were calculated automatically on the Thermal Analysis Universal Analysis 2000 software as a function of temperature. Experiments were performed at least in quadruplicate for each candidate material.

### 2.5. Raman Spectroscopy of IHNs

Raman spectroscopy of the various IHNs was performed using an Avalon Instruments Raman Station R3 (PerkinElmer Life and Analytical Sciences Inc., Wellesley, MA, USA), with a Class 3B laser emitting at 785 nm. Spectra were recorded from 200 to 3200 cm^−1^, with a resolution of 2 cm^−1^ [29].

### 2.6. Attenuated Total Reflectance–Fourier-Transform Infrared Spectroscopy of IHNs

Samples were scanned on a PerkinElmer Spectrum One FT-IR spectrometer (PerkinElmer Life and Analytical Sciences Inc., Wellesley, MA, USA) with a diamond attenuated total reflectance accessory, from 4000 cm^−1^ 750 cm^−1^, with a scan resolution of 4 cm^−1^. A minimum of 32 sub-scans were signal averaged.

### 2.7. Differential Scanning Calorimetry (DSC) of Pluronic Solutions and Hydrated IHNs

DSC was used to investigate the micellization behaviour of the Pluronic block copolymers in solution and to identify micellization in the hydrated IHNs. Solutions for each Pluronic block copolymer (5, 10, and 20% *w*/*v*) were prepared in deionised water and 150 mL of Tris buffer (10% *w*/*w* Pluronic only) and stored at 5 °C. Samples of these solutions (approximately 7 mg) were carefully placed in DSC hermetic aluminium pans and sealed prior to analysis. To investigate micellization in the IHNs, the IHNs were soaked for one week in Tris buffer (pH 7.2), after which samples (approximately 9 mg) removed, and, after excess water was carefully removed from the surface, these were placed in an aluminium hermetic pan and sealed. In both cases, DSC experiments were performed using a TA Instruments Q100 Modulated Differential Scanning Calorimeter (TA Instruments Ltd., Crawley, West Sussex, UK) with a refrigerated cooling system attached. Heat flow was calibrated using an indium standard, and heat capacity with a sapphire standard. Nitrogen was used as the purge gas, flowing at a rate of 50 mLmin^−1^ through the DSC cell. The samples were initially cooled to −10 °C, held isothermally for 10 min and then heated at a rate of 2 °C min^−1^. Micellization was observed in the total heat flow signal as a broad endothermic transition, and the critical micellization temperature (CMT) was accepted as the minimum of the endothermic peak, as determined using Universal Analysis software.

### 2.8. Retention of Pluronic Polymers within IHNs

The IHNs were immersed in 150 mL of Tris buffer (pH 7.2) and at 37 °C with shaking (50 oscillations/min). The samples were removed and placed into a fresh solution at fixed periods, the solvent removed by evaporation, and the residual mass recorded. The mass of the buffer was subtracted from the total mass to enable the mass of Pluronic lost from the IHNs to be determined. The fraction of Pluronic block copolymers released from the IHNs as a function of the theoretical mass of Pluronic block copolymers present was then plotted as a function of time. In all cases, measurements were made on quadruplicate replicate samples.

### 2.9. Statistics

The effects of Pluronic concentration and type on the thermal properties of the IHNs, the critical micelle temperature and the retention of the Pluronic block copolymers within the network were statistically analysed using a two-way analysis of variance (ANOVA) (Prism 10, GraphPad Software). Individual differences between treatments were examined using Dunnett’s test with a probability less than 0.05, denoting significance.

## 3. Results and Discussion

A key challenge to the performance of urinary medical devices is resistance to medical-device-related infection [30]. For example, it has been reported that in 50% of patients who receive urinary catheterisation, infection occurs within 7–10 days [31]. The consequences of catheter-associated infections are considerable, in terms of morbidity/mortality and related healthcare costs [32]. It has been reported (in 2011) that approximately 70% of patients who presented with a urinary tract infection had been catheterised; this occurrence increased to 95% of patients in the intensive care unit setting [33]). Similarly, infection is also a problem for ureteral stents [34]; however, the incidence of infection associated with ureteral stents is lower than with urethral catheters [35]. There have been several proposed strategies to reduce the incidence of urological device-related infection, including changes to device designs (as summarised by [36,37,38]), the materials from which the device is manufactured (polymeric and non-polymeric [37,38,39]), and the use of biodegradable stents [40] and novel coatings, e.g., hydrogels, often containing antimicrobial agents [41,42,43,44,45]. To perform optimally, urological devices/coatings of urological devices must offer a wide range of physicochemical properties, notably mechanical properties and the ability to release a microbicidal concentration of drug at the device–fluid interface for a prolonged period; these properties are rarely achieved using a simple, single polymeric system.

Polymer coatings, e.g., brush coatings, have been shown to offer microbial anti-adherent properties and, as a result, have been proposed to lower the incidence of medical-device-related infection [23]. Several studies have used Pluronic F127, either as the brush coating material or as a brush layer onto which other polymers may be grafted/entrapped, leading to enhanced resistance to microbial colonisation of the device surface [23,24] and blood protein repulsion [25]. These systems most often require a chemical grafting process to locate the Pluronic onto the medical device substrate and then, if required, a further grafting of a second polymer using the Pluronic block copolymer as a substrate. We are currently interested in the entrapment and retention of Pluronics within biomaterials as the inclusion of this polymer may offer modified surface properties, properties that are advantageous as medical device coatings. Accordingly, this study examined the formation of interpenetrating hydrogel networks (IHNs) between three candidate Pluronic block copolymers, offering different physicochemical properties with two model hydrogels, poly(hydroxyethylmethacrylate) and poly(methacrylic acid) [46]. Given the importance of the interactions of the components within the polymer network and the retention of the Pluronic component in aqueous conditions for device performance, this study provides a comprehensive investigation of these key properties, thereby enabling polymer candidates to be identified for further consideration as medical device and/or drug delivery biomaterial.

### 3.1. Thermal and Spectroscopic Properties of Pluronic Block Copolymers

To understand the thermal and spectroscopic properties of IHNs, it was necessary to initially examine the properties of each Pluronic block copolymer. The three grades had the same molecular weight of PPO as the central block (approximately 4000 g mol^−1^). The PEO molecular weight was dependent on the grade of Pluronic tested and consisted of 70% of the total molecular weight for F127, 30% of P123, and only 10% of the total weight for L121. The thermal properties of Pluronic block copolymers used in this study are presented in Table 1. In addition to differences in the melting properties, differences in the degree of crystallinity, associated with the PEO block component of the polymer, were observed, with F127 exhibiting the greatest crystallinity (due to the largest PEO block length). Thus, the observed melting point for F127 was greater than for P123 [47]. Table 1 additionally shows the glass transition temperatures for the three Pluronic block copolymers. The presence of a common glass transition (as opposed to two separate transitions for the PEO and PPO components) located between the glass transition components for the two polymers (−73 °C and −50 °C for PPO and PEO, respectively [48]) indicates that the amorphous phases of PPO and PEO are considerably mixed [49,50]. Reducing the PEO block length significantly reduced the observed glass transition temperature (Tg).

**Figure 1:**
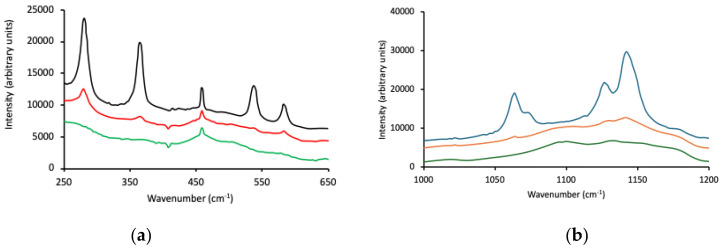
Raman spectra for Pluronic F127 (black line), Pluronic P123 (red line), and Pluronic L121 (green line) within two wavenumber ranges, 250-650 cm^−1^ (**a**) and 1000–1200 cm^−1^ (**b**).

The Raman spectra of Pluronic F127, P123, and L121 over two wavenumber ranges are shown in Figure 1. The region 250–650 cm^−1^ contained peaks attributed to PEO backbone vibrations [51,52], with F127 showing the strongest (highest intensities) and most well-defined peaks of the three block copolymers (Figure 1a). Differences were observed within the spectral region 1000–1200 cm^−1^ as the PEO/PPO ratio decreased (from F127 to P123 to L121, Figure 1b). The bands in the spectrum of F127 (at 1142 cm^−1^ (C–O stretch) and 1064 cm^−1^ (C–O stretch, CH_2_ rock)) were well-defined, whereas the absorbance bands for P123 and L121 were much less distinct and poorly resolved. Strong peaks at 280, 364, 538 and 582 cm^−1^ were detected and assigned to C–C–O, C–O–C bends, and C–C, C–O internal rotations for F127. P123 also showed peaks at these frequencies but were of much lower intensity, whereas peaks for P L121 were not detected at these frequencies. The Raman spectra for Pluronic F127, P123 and L121 are shown in Figure 1a (250–650 cm^−1^) and Figure 1b (1000–1200 cm^−1^).

### 3.2. Thermal and Spectroscopic Properties of IHNs of p(HEMA) and Pluronic Block Co-Polymers

**Figure 2:**
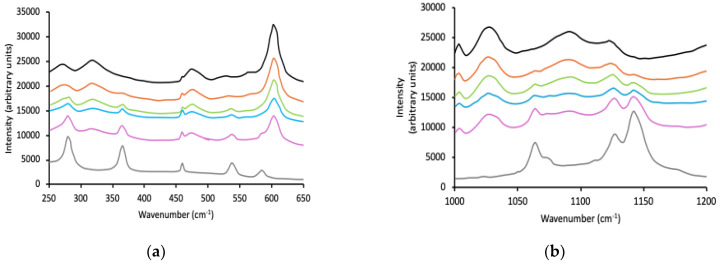

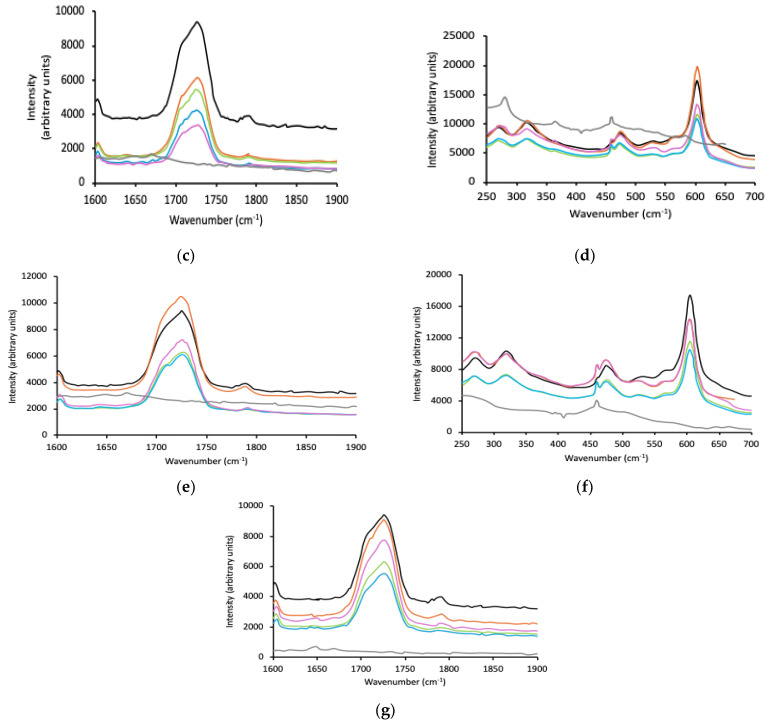
Raman spectra of p(HEMA) (black line), Pluronic (grey line), and p(HEMA)/Pluronic IHNs (10% *w*/*w* red line, 20% *w*/*w*, green line, 30% *w*/*w* blue line, 40% *w*/*w* purple line) within three wavenumber ranges: 250-650 cm^−1^, 1000-1200 cm^−1^ and 1600-1900 cm^−1^. (**a**–**c**) refer to F127, (**d**,**e**) P123 and (**f**,**g**) L121.

Understanding the miscibility (or immiscibility) of the IHNs may be readily performed by consideration of the thermal properties (Table 2) in conjunction with spectroscopic properties (Figure 2). The glass transition temperatures of the IHNs were examined using modulated temperature differential scanning calorimetry and dynamic mechanical analysis, the latter method offering greater sensitivity. Whilst the values for the glass transition of the polymers were greater when measured using DMA (due to the high frequency employed by this method) [53], good correlations were observed between the two methods. The glass transition temperature of p(HEMA) was 114.2 ± 0.6 °C and 126.6 ± 0.5 °C when measured using MTDSC and DMA, respectively, and agrees with previous reports, e.g., [53]. This transition may be attributed to the onset of large-scale, cooperative displacements of the methylene chains of p(HEMA) in the macromolecular network [54].

Incorporation of Pluronic F127, P123, or L121 sequentially from 10% *w*/*w* to 40% *w*/*w* did not change the temperature at which this primary glass transition occurred. Furthermore, a second glass transition was observed with the p(HEMA)/Pluronic IHNs at approximately −67 to −71 °C that was statistically independent of Pluronic concentration, and which was statistically similar to the glass transition temperature of the individual Pluronic components. Accordingly, it may be concluded that each Pluronic is immiscible in the p(HEMA) network; therefore, the p(HEMA)–Pluronic IHNs comprise two distinct phases [54].

The spectroscopic properties of IHNs composed of p(HEMA) and each of the three test Pluronic block copolymers are shown in Figure 2, split into separate wavenumber ranges to enhance clarity. The spectra of p(HEMA)/Pluronic F127 IHNs within the wavenumber ranges of 200–650 cm^−1^, 1000–1200 cm^−1^, and 1600–1900 cm^−1^ are shown in Figure 2a–c, whereas the spectra of p(HEMA)/Pluronic P123 IHNs within the wavenumber ranges of 200–700 cm^−1^ and 1600–1900 cm^−1^ are shown in Figure 2d,e. Finally, the spectra of p(HEMA)/Pluronic L121 IHNs within the wavenumber ranges of 200–700 cm^−1^ and 1600–1900 cm^−1^ are shown in Figure 2f,g. To facilitate analysis, the focus was directed to wavenumbers associated with peaks in p(HEMA) and the Pluronic block copolymers and how these change within the IHNs, notably the carbonyl peak for p(HEMA) (1720 cm^−1^) and the ether peak of PEO (1142 cm^−1^ (C–O stretch) and 1064 cm^−1^ (C–O stretch, CH_2_ rock)). Due to the lower intensity of these vibrations in P123 and L121, greater focus was placed on F127 as potential polymer interactions would be easier to observe. Importantly, it was noted for all three IHNs that no changes in the key wavenumbers associated with the molecular groups of the polymeric components of the IHNs occurred. Similarly, within the fingerprint region of the spectrum (200–700 cm^−1^), the peak at 602 cm^−1^ (associated with C-C-O bond vibration) was present in each of the IHNs at the same wavenumber and the peaks associated with the crystalline regions of F127 (364 and 538 cm^−1^) were detected in the IHNs without any shift in wavenumber. Similarly, the lack of change in the spectra of individual network components has been reported to be evidence of immiscibility in other studies [55,56,57], Accordingly, spectroscopy has provided further evidence of the immiscibility of the two polymers in these IHNs.

### 3.3. Thermal and Spectroscopic Properties of IHNs of p(MAA) and Pluronic Block Co-Polymers

**Figure 3:**
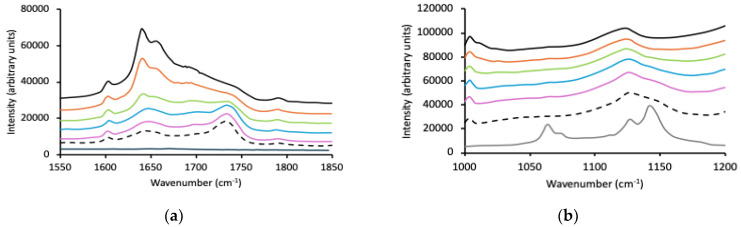

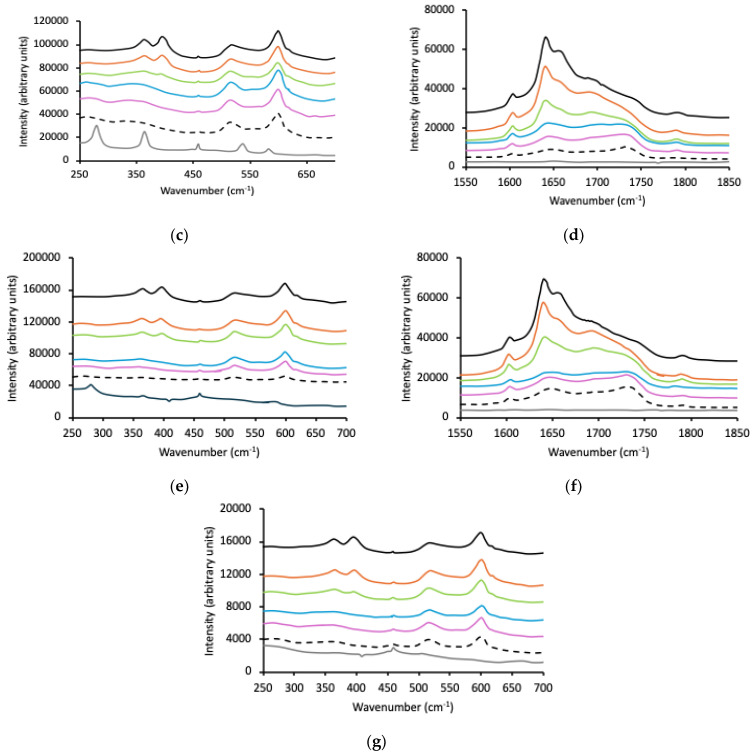
Raman spectra of p(MAA) (black line), Pluronic (grey line), and p(MAA)/Pluronic IHNs (10% *w*/*w* red line, 20% *w*/*w* green line, 30% *w*/*w* blue line, 40% *w*/*w* purple line, 50% *w*/*w* dashed line) within three wavenumber ranges, 250–650 cm^−1^, 1000–1200 cm^−1^, and 1600–1900 cm^−1^; (**a**–**c**) refer to F127, (**d**,**e**) P123 and (**f**,**g**) L121.

The reported applications of p(MAA) as a medical device biomaterial are less than p(HEMA); however, p(MAA) has been used as brushes as biomaterials and for pH-responsive drug delivery applications [58,59,60]. This study aims to understand the interaction between p(MAA) and Pluronic copolymers and their stability within an aqueous environment. Previous studies have confirmed an interaction between poly(acrylic acid, p(AA)) and poly(ethylene oxide). For example, Li et al. [61] described a complexation between p(AA) and PEO that was pH-dependent, and similarly, Monir et al. described an interaction between these two chemical components [62]. As in Section 3.2, the interaction between p(MAA) and Pluronic block copolymers was examined using thermal and spectroscopic methods, presented in Table 3 and Figure 3, respectively.

The glass transition temperature for p(MAA) was 205.5 ± 0.8 °C and 211.5 ± 0.4 °C when measured using MTDSC and DMA, respectively. Sequential addition of Pluronic F127 (0–50% *w*/*w*) resulted in significant successive reductions in the glass transition temperature of p(MAA), and a disappearance of both the glass transition and the melt transition of F127. Reductions in the glass transition temperature of p(MAA) were noted only upon the addition of 30% *w*/*w* of P123 and 30% *w*/*w* L121, with reductions in the main glass transition temperature noted following the addition of 40% *w*/*w* of these block copolymers (although the addition of 50% did not result in a further decrease). There was no statistical difference in the primary glass transitions of p(MAA) containing equivalent concentrations of P123 and L121. At a 50% *w*/*w* loading, the polymer network containing 50% *w*/*w* Pluronic L121 also displayed a secondary glass transition temperature associated with the PPO blocks of the Pluronic component, indicative of phase separation at this concentration. Therefore, it may be concluded that, based on thermal analysis, the IHNs of p(MAA) and Pluronic systems form miscible blends [63,64,65]. The loss of the crystallinity of PEO (and reduction in primary glass transition) suggests an interaction between the PEO component of the Pluronic block copolymers and the carbonyl group of p(MAA) [27,66]. The greater affinity of F127 within the IHNs may be explained by the greater number of PEO blocks.

As before, the spectra of the p(MAA)/Pluronic IHNs have been individually presented in Figure 3 (Figure 3a–c F127, Figure 3d,e P123, and Figure 3f,g L121). p(MAA) typically exhibits a characteristic peak at 1700 cm^−1^ due to the existence of the associated carboxylic acid dimer and a much lower intensity peak at 1730 cm^−1^ due to the presence of the free carbonyl group [67,68,69]. However, these peaks were partially masked by a second peak at 1640 cm^−1^ with a shoulder at 1656 cm^−1^, attributed to the C=C bond of residual methacrylic acid. The Raman spectra of Pluronic block copolymers within the 1500–1850 cm^−1^ region were devoid of peaks. As the concentration of Pluronic F127 was increased within the IHNs, the ratio of the intensity of the shoulder present at 1700 cm^−1^ (associated with the p(MAA) dimer) decreased as the intensity of the shoulder at 1730 cm^−1^ (free carbonyl) increased and resolved into a clear peak. It has been proposed that the miscibility of Pluronic block copolymers within the p(MAA) network is due to a hydrogen bonding interaction between the ether oxygen of PEO and the carboxyl group of p(MAA). Therefore, Figure 3b focuses on the spectral region 1200–1000 cm^−1^, associated with the C-O vibrations in F127. The absence of these peaks in the IHNs, the absence of peaks associated with the the crystallinity of PEO (observed in the wavenumber region 250–650 cm^−1^), and the shift in the wavenumber of the peak due to the carbonyl group of p(MAA) from the dimer to monomer form is evidence of the interaction between the two components in the IHNs. The intensity of the peaks associated with Pluronics P123 and L121 were dramatically lower than that for F127; however, the emergence of the peak at 1730 cm^−1^ was apparent, indicating an interaction with p(MAA).

**Figure 4:**
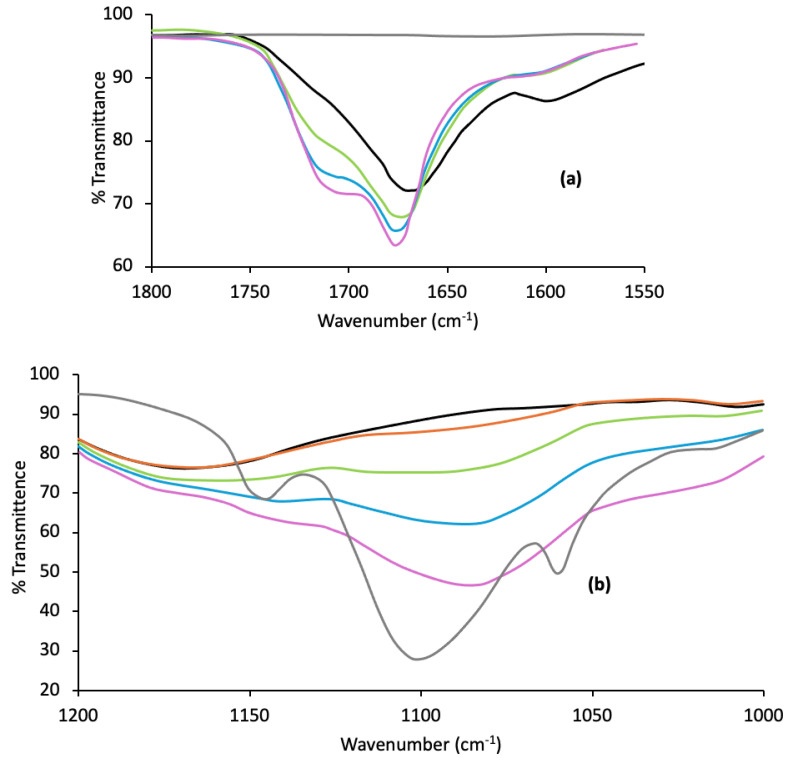
ATR-FTIR spectra of p(MAA) (black line), Pluronic (grey line), and p(MAA)/Pluronic F127 IHNs (10% *w*/*w* red line, 20% *w*/*w* green line, 30% *w*/*w* blue line, 40% *w*/*w* purple line) within two wavenumber ranges, 1550–1800 cm^−1^ (**a**) and 1000–1200 cm^−1^ (**b**).

ATR/FTIR was used to confirm the possible polymer interactions within p(MAA)/P F127 IHNs, with a particular focus on the carbonyl region of the spectra (a region that was partially masked by the C=C vibrational peak in Raman spectroscopy). The spectra for the IHNs with wavenumber ranges of 1550–1800 cm^−1^ and 1000–1200 cm^−1^ are shown in Figure 4a,b, respectively.

A large, broad peak at 1690 cm^−1^ was detected for the p(MAA) homopolymer due to the mainly associated dimeric carbonyl groups. As the concentration of F127 was increased within the network, this peak shifted to a higher wavenumber, indicating the dissociation of the hydrogen bonding between MAA units. With the increasing concentration of F127, a further peak became apparent due to unassociated carbonyl groups at approximately 1730 cm^−1^. The peak at 1100 cm^−1^ in F127 is due to the C-O-C asymmetric stretch, which, in the presence of p(MAA), shifted to a lower wavenumber (circa 1080 cm^−1^, for IHNs containing 30% *w*/*w* and 40% *w*/*w* F127). This indicates the ether group’s involvement in hydrogen bonding with the carbonyl group of p(MAA) [51,70].

### 3.4. Thermal Properties and Loss of Pluronic Block Copolymers from IHNs Following Immersion in Aqueous Fluid

For the IHNs to be clinically successful, either as a medical device biomaterial or as a potential drug delivery platform, the properties of the network should be stable during the period of use. The most potentially catastrophic event would be the rapid loss of the Pluronic block copolymers from the network into the biological fluid, thereby negating any possible advantages of the IHNs. Accordingly, it was important to study the loss of Pluronic block copolymers within the network following the immersion of the IHNs into a simple buffer system (phosphate-buffered saline, pH 7.2) and to identify whether the micellization of the Pluronic block polymers had occurred following immersion in an aqueous environment, the latter phenomenon increasing the effective molecular mass of the Pluronic block copolymers, thereby reducing the diffusion of individual Pluronic monomers from the IHNs. Initially micellization was studied in deionised water and Tris buffer (Table 4).

The temperature at which micellization occurred was dependent on three variables, namely the type and concentration of Pluronic and whether micellization occurred in water or Tris buffer (Table 4). The formation of micelles of the Pluronic copolymers occurs at lower concentrations (CMC) and at lower temperatures (CMT) for Pluronic copolymers with a high PPO:PEO ratio [71]. This is due to the increased hydrophobic interactions in the micelle core compared to Pluronic with a high proportion of PEO. Furthermore, the presence of buffer has been reported to affect the micellization of surfactants.

Micellization was predominantly dependent on the type of Pluronic and the network composition (p(HEMA) and p(MAA)) of the IHNs (Table 5). IHNs containing Pluronic L121 exhibited the lowest critical micelle temperature. Interestingly, micellization was not observed in p(HEMA) IHNs containing Pluronic F127 that had been stored in Tris Buffer for one week.

The losses of each Pluronic block copolymer from p(HEMA)- and p(MAA)-based IHNs over an extended period of storage in Tris buffer are shown in Table 6 and Table 7, respectively. Pluronic loss was statistically dependent on the hydrogel type (p(HEMA) or p(MAA)), the type of Pluronic (F127, P123, L121), and the time of immersion of the IHNs within the buffer. The loss of F127 from the two networks was significant and markedly greater than P123 and L121 and was significantly lower from the p(MAA) network than the p(HEMA) IHNs. Total loss of F127 occurred between 14 and 21 days from the p(HEMA) network, whereas, at equilibrium, the loss of F127 from p(MAA) IHNs ranged from approximately 2% (10% initial loading) to 25% (50% initial loading).

In the IHNs, following the uptake of aqueous fluid, the micellization of the different Pluronic block co-polymers and the dissolution of the Pluronic monomers and subsequent loss from the network in the surrounding aqueous fluid occurred. The retention of various Pluronic block copolymers within the IHNs was facilitated by both the stability/size of the micelle and the interaction of the monomers/micelles with the poly(nethacrylates). The micellization of F127, P123, and L121 did not occur within the IHN xerogels, evidenced by the lack of an endothermic event at or close to the micellization temperature of the various Pluronic block copolymers [72]. As the ratio of PPO to PEO of the Pluronic block copolymers increased ((F127 (0.43) to P123 (2.3) to L121 (9.0)), micellization occurred at lower concentrations due to increased hydrophobic interactions in the micelle core. Pluronic loss was investigated at 37 °C, a temperature that exceeded the critical micelle temperature for each Pluronic block copolymer investigated. It is proposed that the greater retention of Pluronic P123 and L121 within the hydrated IHN is due to their existence within a stable micelle structure, one that resists destabilisation in the presence of aqueous fluid and resists monomer diffusion and loss from the network. The loss of Pluronics from p(MAA) IHNs is further reduced through the chemical interaction of these block copolymers with the acrylate, as evidenced by spectroscopy studies; however, the contribution of this phenomenon is relatively limited in comparison to micellization. Interestingly, the loss of Pluronic P127 from the IHNs was markedly greater than the loss of the other Pluronic block copolymers and, moreover, the differences in loss between the p(MAA) and p(HEMA) IHNs were the most pronounced. In p(HEMA) IHNs, the loss of Pluronic F127 was relatively rapid and micelles were not observed following immersion in Tris buffer for 1 week. This suggests that, whilst F127 micelles may have formed, these were not stable and, as a result, F127 monomer was free to diffuse through the network, leading to loss from the IHNs. The loss of F127 from p(MAA) IHNs was less than from the p(HEMA) IHNs with this stabilising at approximately 4 days. We showed that F127 micelles were present in p(MAA) IHNs following storage in Tris buffer for 1 week, at which point stabilisation of F127 loss had occurred. Therefore, the retention of F127 may be accredited to micelle formation; however, based on the previous spectroscopic observations, hydrogen bonding between p(MAA) and F127 may contribute to micelle stabilisation.

## 4. Conclusions

This manuscript describes novel interpenetrating hydrogel networks (IHNs) composed of p(HEMA) or p(MAA) and different Pluronic block copolymers (F127, P123, and L121) designed for potential use as medical device biomaterials or as platforms for controlled drug delivery. There was minimal interaction with the Pluronic block copolymers in p(HEMA) IHNs, evidenced from Raman spectroscopy and the existence of the individual glass transition temperatures of the components, determined using differential scanning calorimetry (DSC) and dynamic mechanical analysis (DMA). As a result, p(HEMA)-Pluronic block copolymer IHNs may be described as immiscible blends. Conversely, in p(MAA) IHNs, the ether group on the Pluronic block copolymers and the carbonyl group of p(MAA) interacted, as shown from shifts in their wavelength in both Raman spectroscopy and attenuated total reflectance–Fourier–transform spectroscopy. The p(MAA) IHNs exhibited a single glass transition temperature that lay between the two individual network components, the magnitude of which was dependent on the ratio of p(MAA) to Pluronic block polymers. Accordingly, these systems may be described as miscible. The physical state of the Pluronic block polymers within IHN that had been stored in Tris buffer (pH 7.2) for one week was determined using DSC. With the exception of p(HEMA)/F127 IHNs, each Pluronic block copolymer was present as micelles, the critical micelle temperature (CMT) being dependent on the grade of Pluronic block copolymer (decreasing as the ratio of poly(propylene oxide) to poly(ethylene oxide) increased). In the p(HEMA)/F127 IHNs, the micellization of F127 was not observed. Examination of the loss of each Pluronic block copolymer from the two networks following storage in Tris buffer showed that F127 and L121 showed the greatest and lowest loss, respectively. Loss from p(MAA) IHNs was less than from p(HEMA) IHNs. The retention of the Pluronic block copolymers is accredited to their existence as micelles, the intermolecular bonding and large molecular weight of which suppresses Pluronic monomer diffusion and subsequent loss from the network. However, given the greater retention of Pluronic block copolymer p(MAA) IHNs, it may also be concluded that interactions between each Pluronic block copolymer and p(MAA) significantly contributed to the stability of the Pluronics within this network. Based on the observations in this study, notably the stability of several IHNs under aqueous conditions, we are currently assessing these systems as platforms for medical device biomaterials, particularly for use as urinary biomaterials where, if combined with bioactive agents, enhanced resistance to infection may result.

## Figures and Tables

**Table 1:** Glass transition (T_g_), crystallisation (T_c_), and melting (T_m_) temperatures; enthalpies of melting (ΔH_m PEO_) and crystallisation (ΔH_c PEO_) and degree of crystallinity (χ_c PEO_) of Pluronic block copolymers (F127, P123, L121).

Pluronic Grade	Mean (±sd) Glass Transition, Melting and Crystallisation Temperatures			Mean (±sd) Enthalpies of Melting and Crystallinity		Degree of Crystallinity
	T_g_ (°C)	T_m_ (°C)	T_c_ (°C)	ΔH_m,PEO_ (J g^−1^)	ΔH_c,PEO_ (J g^−1^)	χ_c(PEO)_ (%)
P F127	−65.8 ± 0.4	56.5 ± 0.3	Not Measured	116.9 ± 1.4	Not Measured	62.2 ± 0.8
P P123	−68.8 ± 0.4	36.8 ± 0.3	Not Measured	42.8 ± 0.8	Not Measured	22.7 ± 0.4
P L121	−71.4 ± 0.8	Not Measured	−56 ± 0.8	Not Measured	6.6 ± 0.4	Not Measured

**Table 2:** Glass transition temperatures (T_g_), melting points (T_m_), melting enthalpies (ΔH_m_) and degrees of crystallinity (χ_c_) for p(HEMA) and p(HEMA)/Pluronic interpenetrating hydrogel networks (IHNs).

Pluronic Type	Pluronic Conc. (% *w*/*w*)	Mean ± sd Glass Transition Temperature(°C)			Melt Temperature (°C, T_m_)	ΔH_m PEO_ (J g^−1^)
		**T_g_ 1**	**T_g_ 2**	T_g_ 2*		
None	0, p(HEMA)	Not present	114.2± 0.6	126.6 ± 0.5	Not present	Not present
F127	10	Not present	111.3 ± 0.5	126.7 ± 1.5	43.2 ± 1.3	66.0 ± 3.1
	20	−67.0 ± 0.5	112.0 ± 1.1	124.0 ± 1.9	47.5 ± 1.0	85.7 ± 3.4
	30	−67.0 ± 0.5	113.6 ± 0.6	128.1 ± 1.7	50.3 ± 0.9	99.6 ± 3.3
	40	−66.9 ± 0.5	113.9 ± 0.4	128.9 ± 1.1	53.4 ± 0.6	108.9 ± 3.2
	100	−65.8 ± 0.4	Not present	Not present	56.5 ± 0.3	116.9 ± 1.4
P123	10	−67.1 ± 0.4	113.8 ± 0.6	125.8 ± 0.5	24.8 ± 0.7	2.1 ± 0.6
	20	−67.1 ± 0.3	112.7 ± 0.5	124.8 ± 1.4	28.1 ± 0.3	4.8 ± 0.5
	30	−67.4 ± 0.2	112.0 ± 0.7	126.8 ± 0.9	31.7 ± 0.9	8.8 ± 0.8
	40	−67.6 ± 0.3	112.0 ± 0.4	127.5 ± 0.5	33.9 ± 0.8	13.1 ± 0.7
	100	−68.8 ± 0.4	Not present	Not present	36.8 ± 0.3	42.8 ± 0.8
L121	10	−67.9 ± 0.8	114.2 ± 0.6	126.6 ± 0.5	Not present	Not present
	20	−67.9 ± 0.4	113.4 ± 0.5	124.8 ± 2.4	−41.4 ± 0.4	0.2 ± 0.0
	30	−69.0 ± 1.4	113.7 ± 0.8	121.5 ± 1.6	−42.8 ± 0.7	1.0 ± 0.2
	40	−69.0 ± 1.8	113.6 ± 0.9	125.8 ± 1.9	−48.5 ± 0.9	2.4 ± 0.4
	100	−71.3 ± 0.8	113.0 ± 0.9	127.3 ± 1.6	−51.4 ± 0.6	4.5 ± 0.4

Tg 1: Glass transition temperature of the Pluronic component determined using modulated temperature differential scanning calorimetry. Tg 2: Glass transition temperature of the methacrylate component determined using modulated temperature differential scanning calorimetry. Tg 2* Glass transition temperature of the methacrylate component determined using dynamic mechanical analysis.

**Table 3:** Glass transition temperatures (T_g_), melting points (T_m_), melting enthalpies (ΔH_m_), and degrees of crystallinity (χ_c_) for p(MAA), P F127, and p(MAA)/Pluronic inter-penetrating hydrogel networks IHNs.

Pluronic Type	Pluronic Conc. (% *w*/*w*)	Mean ± sd Glass Transition (°C, T_g_)			Melt Temperature (°C, T_m_)	ΔH_m PEO_ (J g^−1^)
		**T_g_ 1**	**T_g_ 2**	T_g_ 2*		
None	0, p(MAA)	Not Present	205.5 ± 0.8	211.5 ± 0.4	Not Present	Not Present
F127	10	Not Present	203.2 ± 1.0	206.8 ± 0.6	Not Present	Not Present
	20		194.8 ± 2.2	195.5 ± 0.8		
	30		171.8 ± 2.0	185.7 ± 0.8		
	40		158.5 ± 2.6	173.1 ± 0.7		
	50		142.3 ± 2.5	168.7 ± 1.5		
	100	−65.8 ± 0.4	Not Present	Not Present	56.5 ± 0.3	116.9 ± 1.4
P123	10	Not Present	208.3 ± 1.7	213.7 ± 0.2	Not Present	Not Present
	20		207.0 ± 0.3	205.3 ± 1.0		
	30		198.5 ± 0.7	199.0 ± 0.5		
	40		193.5 ± 1.0	197.8 ± 0.2		
	50		194.1 ± 1.2	197.3 ± 1.1		
	100	−68.8 ± 0.4	Not Present	Not Present	36.8 ± 0.3	42.8 ± 0.8
L121	10	Not Present	210.0 ± 2.8	213.3 ± 0.5	Not Present	Not Present
	20		207.8 ± 1.0	207.3 ± 0.6		
	30		198.4 ± 1.5	205.9 ± 0.4		
	40		191.9 ± 1.4	202.2 ± 0.2		
	50	−68.4 ± 0.6	189.0 ± 1.5	202.0 ± 0.2		
	100	−71.3 ± 0.8	Not Present	Not Present		4.5 ± 0.4

Tg 1: Glass transition temperature of the Pluronic component determined using modulated temperature differential scanning calorimetry. Tg 2: Glass transition temperature of the methacrylate component determined using modulated temperature differential scanning calorimetry. Tg 2*: Glass transition temperature of the methacrylate component determined using dynamic mechanical analysis.

**Table 4:** Critical micelle temperatures (CMT) of Pluronic solutions in deionised water and Tris buffer.

Concentration of Pluronic (% *w*/*v*)	Mean (±sd) Critical Micellization Temperature of Pluronic Solutions		
	Pluronic F127	Pluronic P123	Pluronic L121
1	not detected	not detected	16.5 ± 0.1
5	25.1 ± 0.1	19.9 ± 0.1	14.4 ± 0.1
10	22.6 ± 0.1	17.4 ± 0.1	13.5 ± 0.1
10 (Tris buffer)	18.3 ± 0.1	14.1 ± 0.1	11.7 ± 0.1
20	17.1 ± 0.2	14.3 ± 0.1	not tested

**Table 5:** The effect of Pluronic type and concentration on their critical micelle temperatures within interpenetrating hydrogel networks (IHNs) that had been stored in Tris buffer (pH 7.2) for 1 week.

Network Composition	Pluronic Grade	Mean (±sd) Critical Micelle Temperature (°C) in IHNs Containing Different Concentrations of Pluronic (% *w*/*w*)					
		0	10	20	30	40	50
p(HEMA)	F127	Not detected	Not detected	Not detected	Not detected	Not detected	Not detected
	P123	Not detected	13.3 ± 1.4	12.8 ± 1.3	11.7 ± 1.4	13.8 ± 1.5	Not determined
	L121	Not detected	11.4 ± 1.2	11.3 ± 1.5	10.5 ± 1.8	9.8 ± 2.1	Not determined
p(MAA)	F127	Not detected	Not detected	18.0 ± 1.2	18.5 ± 1.1	18.9 ± 1.3	19.4 ± 0.9
	P123	Not detected	15.2 ± 1.0	15.8 ± 1.0	15.5 ± 0.7	15.9 ± 0.8	16.0 ± 1.1
	L121	Not detected	12.0 ± 1.1	11.8 ± 1.0	12.4 ± 0.8	12.0 ±0.4	11.9 ± 1.0

**Table 6:** The effect of time of immersion in Tris buffer (pH 7.2) on the % loss of Pluronic block-copolymers within p(HEMA) interpenetrating hydrogel networks (IHNs).

Time (Days)	Mean (±s.d.) % Loss of Pluronic Block Copolymers:											
	Pluronic F127 (% *w*/*w* Initial Loading)				Pluronic P123 (% *w*/*w* Initial Loading)				Pluronic L121 (% *w*/*w* Initial Loading)			
	10	20	30	40	10	20	30	40	10	20	30	40
3	53.4 ± 4.1	73.6 ± 4.9	76.1 ± 5.8	78.1 ± 5.4	0.1 ± 0.0	0.4 ± 0.0	0.8 ± 0.1	2.2 ± 0.0	0.0 ± 0.0	0.9 ± 0.0	1.9 ± 0.2	2.3 ± 0.1
7	87.2 ± 4.0	94.2 ± 7.2	95.2 ± 6.1	96.4 ± 4.9	0.1 ± 0.0	0.5 ± 0.0	2.0 ± 0.3	3.7 ± 0.2	0.1 ± 0.0	1.2 ± 0.1	2.2 ± 0.1	2.7 ± 0.1
14	95.8 ± 5.0	95.0 ± 6.1	94.7 ± 5.4	95.8 ± 6.8	0.1 ± 0.0	1.0 ± 0.0	2.6 ± 0.2	5.5 ± 0.4	0.1 ± 0.0	1.3 ± 0.1	2.2 ± 0.1	3.0 ± 0.2
21	NA	NA	NA	NA	0.2 ± 0.0	1.5 ± 0.2	3.4 ± 0.3	6.4 ± 0.4	0.1 ± 0.0	1.3 ± 0.1	2.3 ± 0.1	3.1 ± 0.3
28	NA	NA	NA	NA	0.3 ± 0.0	2.2 ± 0.2	4.0 ± 0.4	7.5 ± 0.3	0.1 ± 0.0	1.4 ± 0.1	2.4 ± 0.1	3.1 ± 0.2

NA: Not analysed.

**Table 7:** The effect of time of immersion in Tris buffer (pH 7.2) on the % loss of Pluronic block-copolymers within p(MAA) interpenetrating hydrogel networks (IHNs).

Time (Days)	Mean (±s.d.) % Loss of Pluronic Block Copolymers:														
	Pluronic F127 (% *w*/*w* Initial Loading)					Pluronic P123 (% *w*/*w* Initial Loading)					Pluronic L121 (% *w*/*w* Initial Loading)				
	10	20	30	40	50	10	20	30	40	50	10	20	30	40	50
2	1.5 ± 0.1	2.4 ± 0.1	3.1 ± 0.2	9.1 ± 0.4	17.4 ± 2.4	1.2 ± 0.1	2.0 ± 0.1	2.6 ± 0.2	4.5 ± 0.3	9.0 ± 0.5	ND	ND	0.9 ± 0.1	1.3 ± 0.1	2.4 ± 0.1
4	1.8 ± 0.1	3.0 ± 0.2	3.3 ± 0.1	10.9 ± 6.1	24.5 ± 1.8	1.4 ± 0.1	2.6 ± 0.2	3.0 ± 0.1	5.4 ± 0.2	11.9 ± 1.1	0.1 ± 0.0	0.1 ± 0.0	1.1 ± 0.1	1.5 ± 0.1	3.2 ± 0.2
7	2.0 ± 0.1	3.1 ± 0.1	3.5 ± 0.2	13.1 ± 6.2	25.2 ± 1.8	1.6 ± 0.2	2.6 ± 0.2	2.9 ± 0.2	6.2 ± 0.3	14.0 ± 1.4	0.1 ± 0.0	0.1 ± 0.0	1.4 ± 0.1	1.5 ± 0.2	3.7 ± 0.2
14	2.0 ± 0.1	3.2 ± 0.1	3.5 ± 0.2	13.1 ± 2.4	25.3 ± 2.0	1.7 ± 0.2	2.6 ± 0.2	3.0 ± 0.2	6.2 ± 0.4	14.4 ± 1.3	0.1 ± 0.0	0.1 ± 0.0	1.5 ± 0.1	2.0 ± 0.3	3.7 ± 0.3

ND: Not detected.

## Data Availability

Data are contained within the article.
